# Tailoring Amine-Functionalized Ti-MOFs via a Mixed Ligands Strategy for High-Efficiency CO_2_ Capture

**DOI:** 10.3390/nano11123348

**Published:** 2021-12-10

**Authors:** Yinji Wan, Yefan Miao, Tianjie Qiu, Dekai Kong, Yingxiao Wu, Qiuning Zhang, Jinming Shi, Ruiqin Zhong, Ruqiang Zou

**Affiliations:** 1 State Key Laboratory of Heavy Oil Processing, China University of Petroleum-Beijing, No. 18 Fuxue Road, Changping District, Beijing 102249, China; wanyinji0613@163.com (Y.W.); 18332751996@163.com (Y.M.); kongdekai6@163.com (D.K.); 13180275342@163.com (Q.Z.); 2 Beijing Key Laboratory for Theory and Technology of Advanced Battery Materials, School of Materials Science and Engineering, Peking University, No. 5 Yiheyuan Road, Haidian District, Beijing 100871, China; qtjie@pku.edu.cn (T.Q.); yingxiaowucup@163.com (Y.W.); jinmings@pku.edu.cn (J.S.)

**Keywords:** Ti-MOFs, amine functionalization, CO_2_ capture, separation, breakthrough experiment

## Abstract

Amine-functionalized metal-organic frameworks (MOFs) are a promising strategy for the high-efficiency capture and separation of CO_2_. In this work, by tuning the ratio of 1,3,5-benzenetricarboxylic acid (H_3_BTC) to 5-aminoisophthalic acid (5-NH_2_-H_2_IPA), we designed and synthesized a series of amine-functionalized highly stable Ti-based MOFs (named MIP-207-NH_2_-*n*, in which *n* represents 15%, 25%, 50%, 60%, and 100%). The structural analysis shows that the original framework of MIP-207 in the MIP-207-NH_2_-*n* (*n* = 15%, 25%, and 50%) MOFs remains intact when the mole ratio of ligand H_3_BTC to 5-NH_2_-H_2_IPA is less than 1 to 1 in the resulting MOFs. By the introduction of amino groups, MIP-207-NH_2_-25% demonstrates outstanding CO_2_ capture performance up to 3.96 and 2.91 mmol g^−1^, 20.7% and 43.3% higher than those of unmodified MIP-207 at 0 and 25 °C, respectively. Furthermore, the breakthrough experiment indicates that the dynamic CO_2_ adsorption capacity and CO_2_/N_2_ separation factors of MIP-207-NH_2_-25% are increased by about 25% and 15%, respectively. This work provides an additional strategy to construct amine-functionalized MOFs with the maintenance of the original MOF structure and high performance of CO_2_ capture and separation.

## 1. Introduction

More than 85% of the worldwide energy demand is provided by the combustion of fossil fuels [1,2], but at the cost of considerable CO_2_ (3 × 10^13^ kg CO_2_ per year) being emitted into the atmosphere, thus leading to the daunting greenhouse effect [3,4,5]. The carbon capture and storage/sequestration (CCS) technology therefore has been proposed to mitigate emissions of atmospheric CO_2_. For CCS technology, the breakthrough of novel adsorbents with a large CO_2_ working capacity as well as a high CO_2_ selectivity and easy regeneration is the core [6,7,8].

Metal-organic frameworks (MOFs) have been widely used for various applications owing to their ordered crystallinity, high specific surface area, and versatile tunability of chemical environments [9,10,11,12,13,14,15,16,17]. In particular, they can serve as attractive platforms for CO_2_ adsorption and separation to mitigate the greenhouse effect [18,19,20,21,22,23,24,25]. It is widely acknowledged that amine-functionalized MOFs are one of the most effective ways to capture CO_2_, because this method has the advantages of a large working CO_2_ capacity as well as a high CO_2_ selectivity and a low energy penalty for regeneration [7,26]. Currently, many MOF materials have been functionalized by the direct synthesis or post-synthesis modification method to graft amine [27,28,29,30]. For example, Kim et al. [28] prepared a robust tetraamine-functionalized Mg-MOF by the post-synthesis strategy. The tetraamine-functionalized framework showed an excellent CO_2_ trapping efficiency under low CO_2_ partial pressure in the flue stream. Han et al. [31] used a one-step hydrothermal method to synthesize MIL-101(Cr)-NH_2_ nanoparticles. Compared with MIL-101(Cr), the CO_2_ adsorption capacity of MIL-101(Cr)-NH_2_ increased from 1.85 to 2.25 mmol g^−1^. Moreover, the separation factor of CO_2_/N_2_ was enhanced from 7.5 to 11.6 at 1 atm and 35 °C. Recently, Zhong et al. [32] introduced three kinds of organic amine molecules into the channels of MIL-101(Cr) by the post-modification method. The results showed that the CO_2_/CO selectivity of the Tris(2-aminoethyl) amine-modified MIL-101 was 103 times higher than that of its pure MIL-101 counterpart.

**Figure 1:**
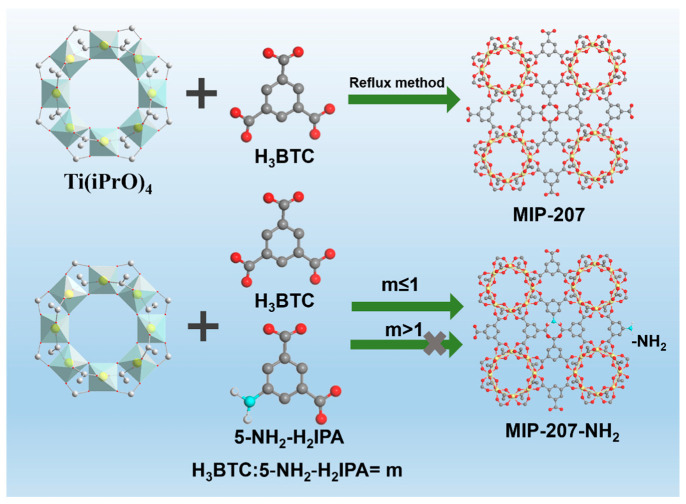
Schematic Diagram of MIP-207 and amine-functionalized MIP-207; Ti is shown in yellow, C in gray, O in red, N in light blue, and H in white.

In this work, the MIP-207, fabricated by Ti–O clusters and the H_3_BTC ligand [21,33], was selected as the porous material, mainly due to its large specific surface area and high chemical stability even in highly acidic media (pH ≤ 0). More importantly, there are uncoordinated and isolated -COOH groups toward the channels of MIP-207 because of the meta-connection mode of the H_3_BTC ligand, so the chemical environment of MIP-207 cavities can be easily modulated by the mixed linkers strategy [33]. There are quite a few reports on amine-grafted highly stable MIP-207 for highly efficient CO_2_ capture [33]. Additionally, the accurate grafting of amine molecules without any framework destruction of the host frameworks is less of a concern. Herein, we for the first time prepared a series of amine-functionalized highly stable MIP-207 materials and further tailored the content of -NH_2_ by the mixed linkers strategy for capturing CO_2_ from N_2_. It should be pointed out that amine-functionalized MIP-207 materials cannot be obtained when the mole ratio of H_3_BTC to 5-NH_2_-H_2_IPA exceeds 1 (See Figure 1). The physiochemical properties of the as-prepared materials were systematically characterized and analyzed, and the CO_2_ adsorption and separation were also investigated. Based on the excellent CO_2_ capture performance of MIP-207-NH_2_-25%, the breakthrough experiments further evaluated the dynamic adsorption capacity and separation factors under different gas flow rates.

## 2. Experimental Section

### 2.1. Synthesis of MIP-207

All the reagents used were commercially purchased without further purification. MIP-207 was synthesized in a similar method to the one reported [33]. 1,3,5-benzenetricarboxylic acid (H_3_BTC), acetic acid, and acetic anhydride were purchased from Maclin, Shanghai, China. Tetraisopropyl titanate was purchased from Sinopharm Chemical Reagent Co., Ltd., Beijing, China. H_3_BTC (840 mg, 4 mmol), acetic acid (10 mL) and acetic anhydride (10 mL) were added and mixed into a 50 mL round-bottom flask at ambient temperature. Then, tetraisopropyl titanate (800 μL, 2.7 mmol) was added under stirring. The mixture was refluxed at 120 °C for 12 h. After cooling to room temperature, the crude product was separated and washed with boiling anhydrous acetone. Finally, the product was collected by centrifugation and placed in an 80 °C oven for 12 h.

### 2.2. Synthesis of Amine-Functionalized MIP-207

The amine-functionalized MIP-207 was prepared with a pre-synthesis modification method. Part of the H_3_BTC ligand was replaced with a certain amount of 5-NH_2_-H_2_IPA, which accounted for 15%, 25%, 50%, 60%, and 100% of the total H_3_BTC, respectively. A series of amine-functionalized MIP-207 materials were synthesized according to the above synthetic steps of MIP-207, and the as-prepared materials were denoted as MIP-207-NH_2_-*n* (*n* = 15%, 25%, 50%, 60%, and 100%), respectively. It must be pointed out that the structure of MIP-207-NH_2_-100% is completely different from that of MIP-207; it is still named MIP-207-NH_2_-100% simply for the purpose of comparison.

### 2.3. Sample Characterization

Powder X-ray diffraction (PXRD) patterns of the samples were recorded on a Rigaku D/max 2400 X-ray diffractometer equipped with Cu K_α_ radiation operating at 45 kV and 200 mA. Scanning electron microscopy (SEM) tests were conducted on a Hitachi S4800 electron microscope to observe the morphologies of the samples. A N_2_ physisorption test was carried out on a Quantachrome Autosorb-iQ (Quantachrome Instruments, Boynton Beach, FL, USA) at −196 °C. The elemental analysis (EA) of the samples was performed on an Elementar Analysensysteme GmbH Vario EL (Analytical Instrumentation Department of the Heraeus technology group, Frankfurt, Germany) analyzer to accurately analyze the percentage content of the C, H, and N elements of samples. The specific surface area of the samples was analyzed according to the Brunauer-Emmett-Teller (BET) method and pore size distribution was calculated using the non-local density functional theory (NLDFT) model.

### 2.4. Gas Adsorption Measurements

All the gases (N_2_ and CO_2_) used were of ultrahigh purity (99.999%) in this study. N_2_ and CO_2_ adsorption isotherms were measured by a Quantachrome Autosorb-iQ gas adsorption analyzer up to 1 bar, and the temperatures of 0 and 25 °C were both maintained with an ethylene glycol/H_2_O bath by a cooling and heating system. Before the measurement, about 100 mg of the adsorbent was degassed at 150 °C for 8 h in vacuum condition. The adsorption and desorption of CO_2_ cyclic stability was carried out on an SDT Q600 analyzer (TA Instruments, New Castle, DE, USA). Firstly, the sample fully absorbed CO_2_ at 35 °C for 1 h, and then it was injected with N_2_ gas at 150 °C for 2 h. The breakthrough experiments were performed on a homemade setup to simulate the actual mixture gas (20 vol% CO_2_, 20 vol% N_2_, and balanced gas He) separation to evaluate the dynamic CO_2_/N_2_ adsorption performance; the setup diagram of the breakthrough experiment can be found in our previous work [34].

## 3. Results and Discussion

### 3.1. Structural Analysis of Samples

**Figure 2:**
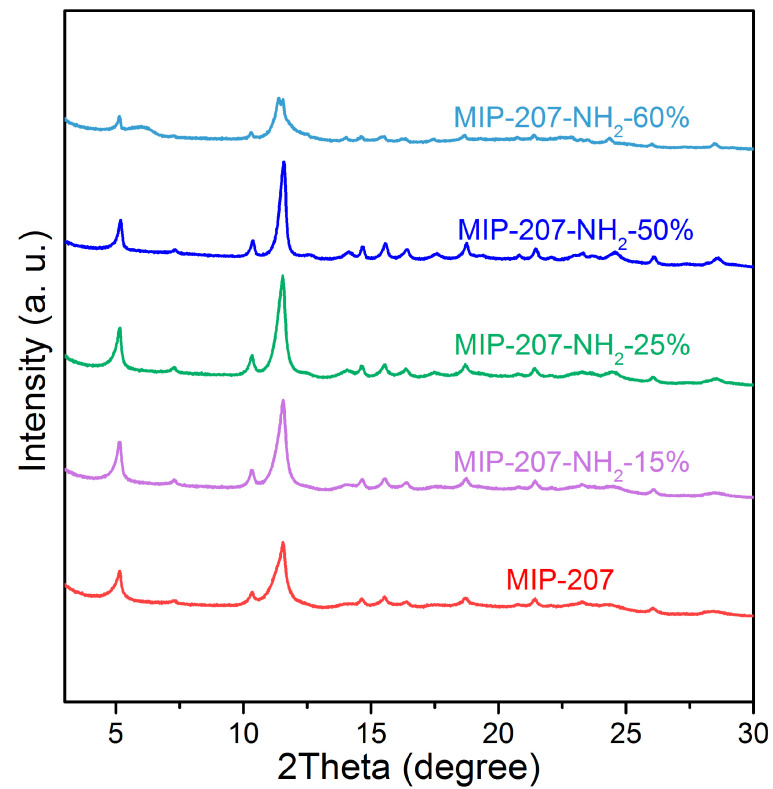
PXRD pattern of samples.

As shown in Figure S1, compared with simulated MIP-207, the characteristic diffraction peak positions and relative intensities of MIP-207 after reflux treatment at 120 °C for 12 h fit very well, illustrating that MIP-207 with a high purity was synthesized. Also, Figure S1 exhibits that the structure of MIP-207 was maintained well after activation at 150 °C. To evaluate the influence of the content of -NH_2_ on the crystal structure of MIP-207, the PXRD patterns of MIP-207-NH_2_ were obtained, and the results are shown in Figure 2. The comparison of PXRD patterns among 207-NH_2_-*n* (*n* = 0, 15%, 25%, 50%) supported that they were of the same pure phase. However, the internal crystal structure of the amine-modified MIP-207 composites started to change when the mole ratio of H_3_BTC to 5-NH_2_-H_2_IPA was more than 1. As shown in Figure 2, there were still two characteristic diffraction peaks of 5° and 11.5° in the MIP-207-NH_2_-60%, but peak relative intensities were significantly reduced, showing that most of the crystal structure of MIP-207 in the composites was changed. When the ligand H_3_BTC was totally replaced by 5-NH_2_-H_2_IPA, the characteristic diffraction peaks (Figure S2) of the sample were totally different from those of the original MIP-207, indicating that another crystalline phase was formed due to the transformation of the coordination mode.

The SEM images of pristine MIP-207 are presented in Figure S3a,b, and the stacked nanoparticles with a size range of 20–25 nm can be observed. As shown in Figure S3c–e, as the amount of exchange ligand 5-NH_2_-H_2_IPA increases, the stacking of nanoparticles becomes loose, and the particle size was also in the range of 20–25 nm in the MIP-207-NH_2_-*n* (*n* = 15%, 25%, 50%) composites, which is consistent with XRD results obtained by the Scherrer equation (Table 1). However, the original morphology of MIP-207 is basically not observed in the MIP-207-NH_2_-60% (Figure S3f), and particle size sharply reduced to about 15 nm. Overall, based on the above PXRD and SEM analysis, the crystal structure and texture of MIP-207 in the amine-modified MIP-207 composites can be maintained with the amount of added 5-NH_2_-H_2_IPA being less than or equal to 50%.

As shown in Table 1, the N element was not found in the parent MIP-207. The N element was detected and the N content of the amine-modified MIP-207 composites increased with the increase of the added 5-NH_2_-H_2_IPA ligand, demonstrating that -NH_2_ was introduced into the framework of MIP-207 through a mixed linkers strategy. Notably, Table 1 indicates that the N element content in the MIP-207-NH_2_-*n* (*n* = 15%, 25%, 50%) composites was lower than the theoretical value, which is attributed to the electronic effect of the functional group of the ligand. Generally, the electron-donating groups such as -NH_2_, -OH, and -CH_3_ are difficult to connect with the second building units of MIP-207 [33]. The theoretical N content of MIP-207-NH_2_-100% is 3.54%. This value is close to the actual value, while the structure completely changed according to the PXRD results.

**Figure 3:**
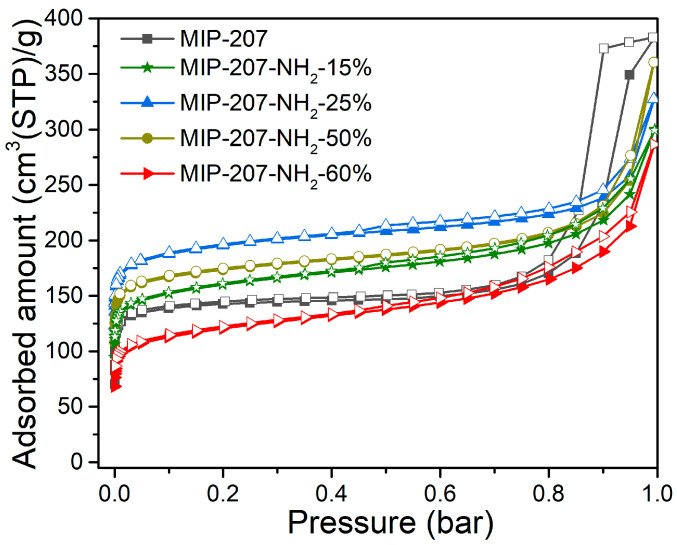
N_2_ adsorption and desorption isotherms at −196 °C.

The results of the measurement of N_2_ adsorption and desorption isotherms are shown in Figure 3 and Table 2, and the N_2_ adsorption–desorption curves of MIP-207 (Figure 3) conform to the typical I-type isotherm characteristics in the low-pressure zone (0–0.6 atm) relating to microporous characteristics [35]. With the increase of pressure, there was a hysteresis loop in the adsorption–desorption curves, indicating the existence of mesopores, which may be caused by the accumulation of materials. The specific surface area of MIP-207 was 563 m^2^ g^−1^, where the specific surface area was mainly micropores (534 m^2^ g^−1^), which confirms that the mesopores are caused by stacked pores. Figure S4 shows that the average pore size of MIP-207 was mainly distributed at 0.57 and 0.82 nm. Obviously, the BET area and pore volume of the MIP-207-NH_2_-*n* (*n* = 15%, 25%, 50%) materials were higher than that of the unmodified MIP-207 (Table 2), mainly because the mass and volume of the -NH_2_ group is smaller than the -COOH group, so the BET area of MIP-207-NH_2_-*n* (*n* = 15%, 25%, 50%) increased. Among them, the BET area of MIP-207-NH_2_-25% was the highest, reaching 735 m^2^ g^−1^. On the contrary, the BET area of MIP-207-NH_2_-60% reduced in comparison with MIP-207. In addition, compared with the MIP-207-NH_2_-*n* (*n* = 15%, 25%, 50%) (Table 2), the pore volume of MIP-207-NH_2_-60% (0.44 cm^3^ g^−1^) decreased. The probable reason is that the original structure of MIP-207 cannot be maintained with the -NH_2_ increasing to over 60%. The specific surface area of MIP-207-NH_2_-100% sharply decreased and the micropores almost disappeared (Figure S5 and Table S1), further demonstrating the changes from the MIP-207 framework in MIP-207-NH_2_-100%.

### 3.2. Gas Adsorption Performance of Materials

**Figure 4:**
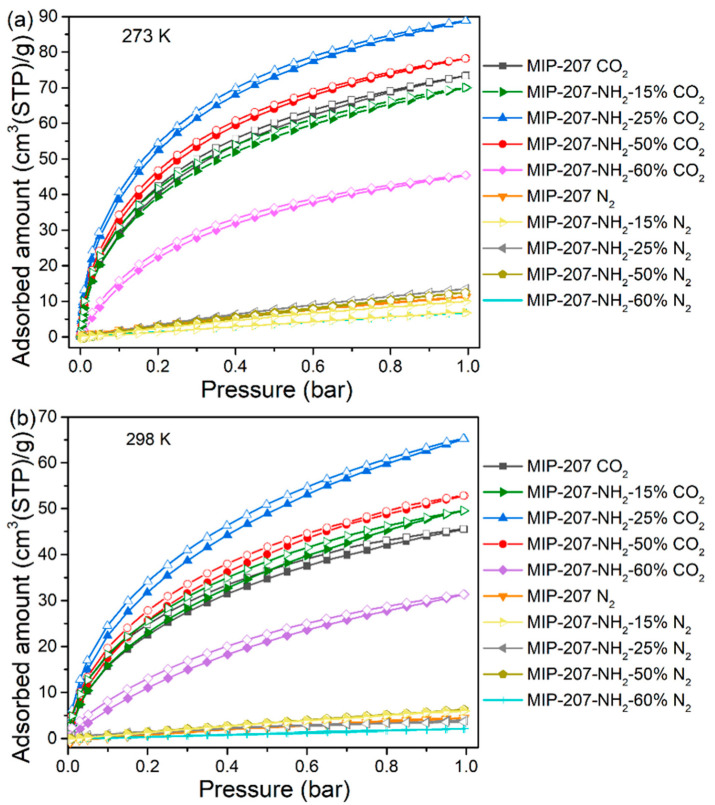
CO_2_ and N_2_ adsorption and desorption isotherms at (**a**) 0 °C and (**b**) 25 °C.

The CO_2_ adsorption data of as-prepared materials over N_2_ are presented in Figure 4 and Table 3. As can be seen from Table 3, the CO_2_ adsorption capacity of MIP-207-NH_2_-25% was up to 3.96 and 2.91 mmol g^−1^ at 0 and 25 °C, which means an improvement of 20.7% and 43.3% compared with the pure MIP-207, respectively. Moreover, the CO_2_ capture performance of MIP-207-NH_2_-25% outperforms most reported amine-modified MOF CO_2_ adsorbents (Table 3). Similarly, the CO_2_ adsorption capacity of MIP-207-NH_2_-50% was higher than that of the unmodified MIP-207. The increase of CO_2_ adsorption capacity is mainly due to the amine-grafted MIP-207 materials with a high specific area (Figure S6) and many Lewis basic sites (LBS), which greatly enhance their affinity for CO_2_ [36,37]. Unfortunately, as the added exchange ligand 5-NH_2_-H_2_IPA went above 50%, the CO_2_ working capacity in the MIP-207-NH_2_-60% adsorbent sharply decreased. One reasonable explanation is that excess 5-NH_2_-H_2_IPA slows down the rate of the crystal nucleation formation of MIP-207 and disturbs the self-assembly process. When the ligand reactant is completely 5-NH_2_-H_2_IPA, the resulting product cannot even form the original crystal nucleus structure of MIP-207. It can be seen that the adsorption performance is a result of both the adsorption sites and the spatial framework of materials.

**Figure 5:**
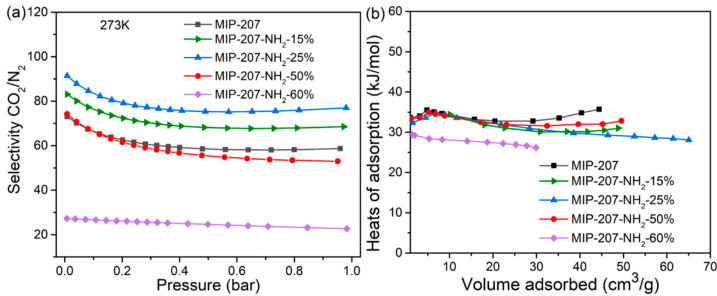
(**a**) CO_2_/N_2_ selectivity at 0 °C and (**b**) CO_2_ adsorption enthalpy curves.

**Figure 6:**
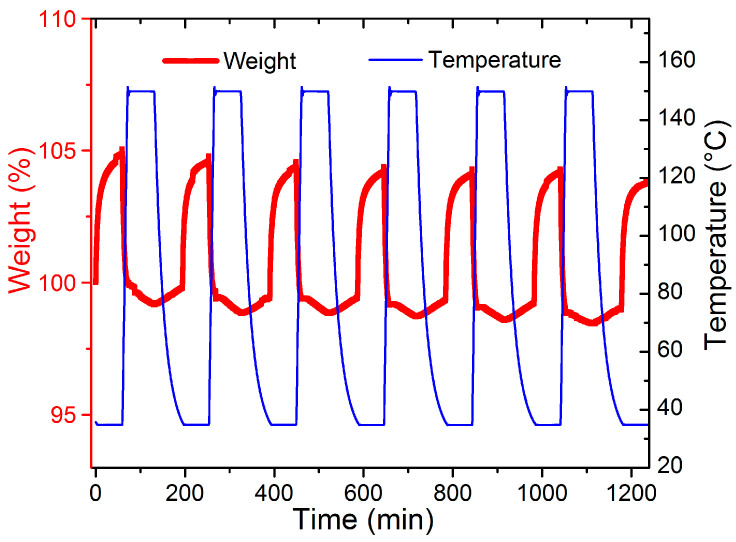
CO_2_ adsorption and desorption cycle of MIP-207-NH_2_-25%.

To figure out the CO_2_ adsorption separation performance, the selectivity of CO_2_/N_2_ was calculated by the IAST model (Supporting Information). As shown in Figure 5a, compared with the separation factor of MIP-207 (59), the CO_2_/N_2_ separation factor of MIP-207-NH_2_-15% was 69, which was 17% higher than that of MIP-207. Additionally, MIP-207-NH_2_-25% exhibited the highest CO_2_/N_2_ separation factor (77), which was 33% higher than MIP-207, mainly because the introduction of the -NH_2_ group into the MIP-207 channels could produce more adsorption sites, leading to an enhanced affinity toward CO_2_. However, the structure of MIP-207-NH_2_-60% possessed more -NH_2_ and the separation factor of MIP-207-NH_2_-60% was only 22, illustrating that the spatial framework of MIP-207 in MIP-207-NH_2_-60% was destroyed, thus increasing the non-selective uptake. In addition to focusing on the adsorption and separation performance, it is also necessary to take energy consumption into account during the regeneration process in industrial applications [32]. The isosteric heat of CO_2_ adsorption (Q_st_) of the materials was obtained from the CO_2_ adsorption isotherms at 0 °C. As shown in Figure 5b, the MIP-207-NH_2_-*n* (*n* = 15%, 25%, 50%) adsorbents had an isosteric adsorption heat of about 30–35 kJ mol^−1^, which exhibits a medium-strength interaction with CO_2_. It can also be found that the Q_st_ of MIP-207-NH_2_-60% was significantly lower than that of the above four materials, which indicates that the framework structure of MIP-207-NH_2_-60% has a negative effect on the adsorption capacity of CO_2_. Considering that the MIP-207-NH_2_-25% exhibited superior CO_2_ adsorption performance with a remarkable adsorption heat, a test of the cyclic stability of CO_2_ was performed and the results are displayed in Figure 6. The CO_2_ adsorption capacity of MIP-207-NH_2_-25% after six cycles did not significantly decrease, indicating that MIP-207-NH_2_-25% has an outstanding CO_2_ adsorption–desorption stability.

**Figure 7:**
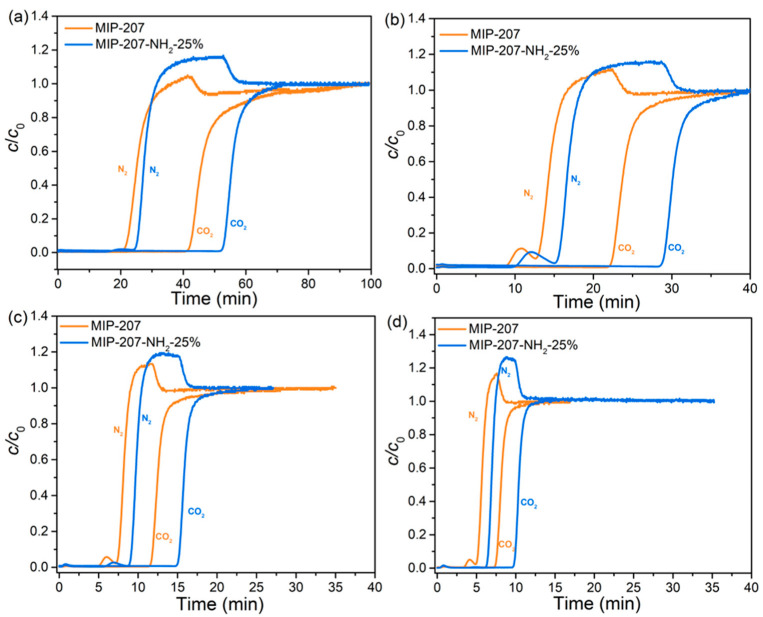
CO_2_ and N_2_ breakthrough curves of MIP-207 and MIP-207-NH_2_-25% at different gas flow rates: (**a**) 10 sccm, (**b**) 20 sccm, (**c**) 50 sccm, and (**d**) 100 sccm, respectively.

The results of the dynamic CO_2_/N_2_ adsorption of both MIP-207-NH_2_-25% and MIP-207 (as a comparison) are shown in Figure 7. There was an obvious difference in breakthrough time between CO_2_ and N_2_ under different gas flow rates. After an initial period where the N_2_ and CO_2_ were fully absorbed, the N_2_ preferentially penetrated the adsorption bed, followed by the CO_2_. The outlet concentration of N_2_ exceeded the inlet concentration because CO_2_ adsorption equilibrium was not reached. Finally, CO_2_ began to be eluted and the concentration of N_2_ and CO_2_ gradually reached the feed concentration value (*c/c*_0_ = 1), indicating that the adsorption bed was saturated. The interval of breakthrough time between CO_2_ and N_2_ in MIP-207-NH_2_-25% was longer than that of MIP-207 at any gas flow rate, especially at 10 sccm, which fully demonstrates that MIP-207-NH_2_-25% has a better dynamic separation performance. The reason is that the electric field of the MIP-207-NH_2_-25% framework has the stronger interaction with CO_2_ due to the presence of LBSs and the hydrogen bond [37,39,40,41]. Moreover, from Figure 7, MIP-207-NH_2_-25% has a larger slope than MIP-207 under different gas flow rates, indicating that the mass transfer resistance of gas in MIP-207-NH_2_-25% is smaller, which is more conducive to gas diffusion and spread. The possible reason for this is that MIP-207-NH_2_-25% has a larger pore volume.

**Figure 8:**
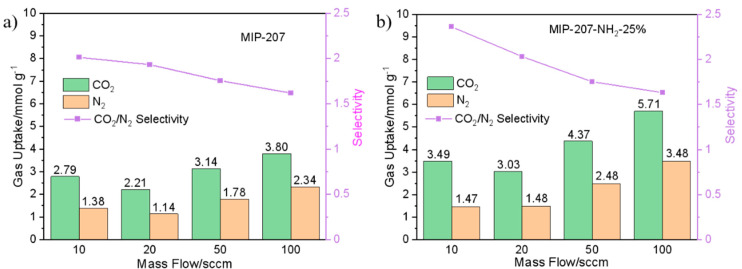
Dynamic CO_2_ and N_2_ adsorption and separation performance of (**a**) MIP-207 and (**b**) MIP-207-NH_2_-25%.

The dynamic equilibrium adsorption capacity and separation factor of both MIP-207 and MIP-207-NH_2_-25% were calculated based on previous reports [34,42]. As shown in Figure 8, it can be found that the CO_2_ equilibrium adsorption capacity of MIP-207-NH_2_-25% was higher than that of MIP-207 under the four mixed gas flow rates (Figure 8a,b). At 10 and 20 sccm, the CO_2_/N_2_ separation factors of MIP-207-NH_2_-25% were 2.36 and 2.03, which were higher than the 2.01 and 1.93 of MIP-207, respectively. The difference of separation factors between MIP-207 and MIP-207-NH_2_-25% cannot be clearly observed at the mixture gas flow rates of 50 and 100 sccm (Figure 8a,b). This is because the residence time of gas in the adsorption bed decreases with the increase of flow rate.

## 4. Conclusions

In summary, the amine-modified highly stable MIP-207 with different -NH_2_ content was successfully prepared by the mixed linkers method. The texture and structure framework of the original MIP-207 were maintained in the MIP-207-NH_2_-*n* (*n* = 15%, 25%, 50%) composites. The CO_2_ adsorption and breakthrough experiments show that MIP-207-NH_2_-25% demonstrates the superior CO_2_ capture and separation performance. The highly efficient CO_2_ uptake is attributed to the introduction of -NH_2_ into the framework of MIP-207, leading to the increase of specific surface area and more Lewis basic adsorption sites, thereby enhancing the CO_2_ working capacity and CO_2_/N_2_ selectivity. This work provides an additional avenue to prepare highly stable amine-functionalized MOFs for high efficiency CO_2_ capture.

## Figures and Tables

**Table 1:** The content of C, H, and N elements of samples and particle size obtained by Scherrer equation.

Samples	C (%)	H (%)	The Actual N (%)	The Theoretical N (%)	Particle Size (nm)
MIP-207	35.37	2.91	0	0	21.9
MIP-207-NH_2_-15%	35.46	2.76	0.19	0.47	22.3
MIP-207-NH_2_-25%	35.03	2.72	0.26	0.83	23.6
MIP-207-NH_2_-50%	35.07	2.89	0.45	1.70	28.4
MIP-207-NH_2_-60%	35.11	3.04	0.93	2.06	15.5
MIP-207-NH_2_-100%	41.48	3.99	3.24	3.54	-

Note: The theoretical value of the N element is calculated assuming that 5-NH_2_-H_2_IPA completely reacts.

**Table 2:** The summary of specific surface area, pore volume, and particle size of samples.

Samples	BET Area (m^2^ g^−1^)	Micropore Area (m^2^ g^−1^)	Total Pore Volume (cm^3^ g^−1^)	Micropore Volume (cm^3^ g^−1^)
MIP-207	563	534	0.36	0.21
MIP-207-NH_2_-15%	576	468	0.46	0.20
MIP-207-NH_2_-25%	735	659	0.51	0.27
MIP-207-NH_2_-50%	654	569	0.56	0.23
MIP-207-NH_2_-60%	435	321	0.44	0.14

**Table 3:** The summary of BET area and CO_2_ adsorption results in this work and reported amine-functionalized MOFs.

Materials	Surface Area (m^2^ g^−1^)	CO_2_ Uptake at Testing Condition	CO_2_/N_2_ (CO) Selectivity	Q_st_ (kJ mol^−1^)	Ref.
MIP-207	563	3.28/2.03 mmol g^−1^ @ 0/25 °C and 1 bar	59	-	This work
MIP-207-NH_2_-15%	576	3.12/2.21 mmol g^−1^ @ 0/25 °C and 1 bar	-	30–35	This work
MIP-207-NH_2_-25%	735	3.96/2.91 mmol g^−1^ @ 0/25 °C and 1 bar	77	30–35	This work
MIP-207-NH_2_-50%	654	3.49/2.36 mmol g^−1^ @ 0/25 °C and 1 bar	-	30–35	This work
MIP-207-NH_2_-60%	435	2.02/1.04 mmol g^−1^ @ 0/25 °C and 1 bar	-	30–35	This work
ZIF-8 (40)	844	0.11 mmol g^−1^ @ 45 °C and 0.15 bar	-	55	[19]
ED@Cu_3_(BTC)_2_-1	444	4.28/2.15 mmol g^−1^ @ 0/25 °C and 1 bar	21.5	39	[29]
ED@Cu_3_(BTC)_2_-2	163	1.03/0.54 mmol g^−1^ @ 0/and 1 bar	2.68	-	[29]
MAF-23	-	2.5 mmol g^−1^ @ 25 °C and 1 bar	87	34.9 ± 0.9	[38]
ED@MIL-101	1584.6	3.93/1.93 mmol g^−1^ @ 0/25 °C and 1 bar	17.3	-	[32]
TEDA@MIL-101	1806.9	3.81/1.65 mmol g^−1^ @ 0/25 °C and 1 bar	15.5	-	[32]
MIL-101(Cr)-NH_2_	2800 ± 200	3.4 mmol g^−1^ @ 15 °C and 1 bar	26.5	54.6	[31]
PM24@MOF	2550	2.9 mmol g^−1^ @ 0/25 °C and 1 bar	84	84	[39]
R-PM24@MOF	2410	3.6 mmol g^−1^ @ 0/25 °C and 1 bar	143	50	[39]

## Data Availability

All data are available upon reasonable request.

## Supplementary Materials

The following are available online at https://www.mdpi.com/article/10.3390/nano11123348/s1, Figure S1: PXRD patterns of MIP-207 and MIP-207 after activation at 150 °C. Figure S2: PXRD pattern of MIP-207-NH_2_-100%. Figure S3: SEM images of (**a**,**b**) MIP-207, (**c**) MIP-207-NH_2_-15%, (**d**) MIP-207-NH_2_-25%, (**e**) MIP-207-NH_2_-50%, and (**f**) MIP-207-NH_2_-60%. Figure S4: The pore size distribution curves of MIP-207. Figure S5: N_2_ adsorption and desorption isotherms of MIP-207-NH_2_-100%. Figure S6: The relationship of between CO_2_/N_2_ adsorption and specific surface area of samples. Table S1: The BET data of MIP-207-NH_2_-100%.
