# Supplementary material for: Tailoring Amine-Functionalized Ti-MOFs via a Mixed Ligands Strategy for High-Efficiency CO2 Capture
Source: Nanomaterials (Basel). 2021 Dec 10;11(12):3348. doi: 10.3390/nano11123348 (PMC8709442; doi:10.3390/nano11123348)
Supplement: Supplementary file 1 [file nanomaterials-11-03348-s001.zip › nanomaterials-1477209-supplementary.pdf]

Tailoring Amine-Functionalized Ti-MOFs via a Mixed Ligands Strategy for High-Efficiency CO_2 Capture

Yinji Wan [1,†], Yefan Miao [1,†], Tianjie Qiu [2], Dekai Kong [1], Yingxiao Wu [2], Qiuning Zhang [1], Jinming Shi [2], Ruiqin Zhong [1,*] and Ruqiang Zou [2,*]

1 State Key Laboratory of Heavy Oil Processing, China University of Petroleum, Beijing, No. 18 Fuxue Road, Changping District, Beijing 102249, China; wanyinji0613@163.com (Y.W.); 18332751996@163.com (Y.M.); kongdekai6@163.com (D.K.); 13180275342@163.com (Q.Z.)

2 Beijing Key Laboratory for Theory and Technology of Advanced Battery Materials, School of Materials Science and Engineering, Peking University, No. 5 Yiheyuan Road, Haidian District, Beijing 100871, China; qtjie@pku.edu.cn (T.Q.); yingxiaowucup@163.com (Y.W.); jinmings@pku.edu.cn (J.S.)

* Correspondence: zhong2004@foxmail.com (R.Z.); rzou@pku.edu.cn (R.Z.)

† These authors contributed equally to this work.

Selectivity calculations of ideal adsorbed solution theory (IAST)

The ideal adsorbed solution theory (IAST) was employed to estimate the CO_2/N_2 separation performance through the single component sorption isotherms. The CO_2 and N_2 adsorption isotherms were fitted to the dual-sites Langmuir-Freundlich model (Eq. S1). In this work, the IAST selectivity was calculated based on a CO_2/N_2 (15 vol%:85 vol%) binary mixed gas at 273 K and pressures up to 1 bar to mimic the composition and condition of flue gas. The detailed calculating procedure of the IAST method has been reported in many works [1-3] .

$$\mathrm{V} = \frac{\mathrm{V}_1 K_1 P^{n_1}}{1 + K_1 P^{n_1}} + \frac{\mathrm{V}_2 K_2 P^{n_2}}{1 + K_2 P^{n_2}} \quad (Equation\ S1)$$

Where V_1, V_2 are adsorbed gas capacity; K_1, K_2 are calculated coefficients; P is the gas pressure (bar); and n_1, n_2 represent the deviation from the ideal uniform surface. According to the IAST, the adsorbed gas can be regarded as two phases. The chemical potential of each component in adsorbed phase must be equal to that of each component in the gas phase. If the intermolecular force is neglected, the relationship of one component in the two phases satisfy the Raoul's Law when the system reaches equilibrium:

$$p_i = p y_i = p_i^0 x_i \quad (Equation\ S2)$$

Where is p_i is the partial pressure of i component (bar); P is the gas phase pressure (bar); y_i is the mole fraction of i component in the gas phase; p_i^0 is equilibrium vapor pressure (bar). and x_i is the mole fraction of i component in the adsorbed phase. According to the Gibbs equation, the following equation can be obtained:

In IAST, p_i^0 is defined by relating to spreading pressure π,

$$\frac{\pi \mathrm{A}}{RT} = \int_0^{p_i^0} \frac{n}{P} dP \quad (Equation\ S3)$$

π is spreading pressure, A is specific surface area of adsorbent ($m^2\ g^{-1}$), R is gas constant (8.314 $J\ K^{-1}\ mol^{-1}$), T is temperature (K). In equilibrium, the surface tension of each component in adsorptive phase should be equal, and

the sum of the mole fractions of each component should be 1.

$$\int_0^{p_i^0} \frac{n_i}{P} dP = \int_0^{p_j^0} \frac{n_j}{P} dP \quad (i, j = 1 \dots m) \quad (Equation\ S4)$$

$$\sum_{i=1}^{2} x_i = 1 \quad (Equation\ S5)$$

When the isotherm equation is introduced into equation (S3) (S4) (S5), the relationship between pressure and adsorption capacity of each component can be obtained, which can be used to calculate selectivity:

$$Selectivity = \frac{x_i/y_i}{x_j/y_j}$$

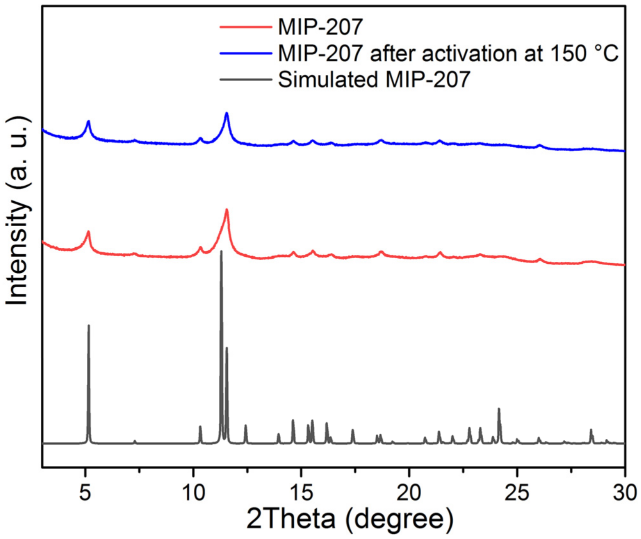

Figure S1. PXRD patterns of MIP-207 and MIP-207 after activation at 150 °C.

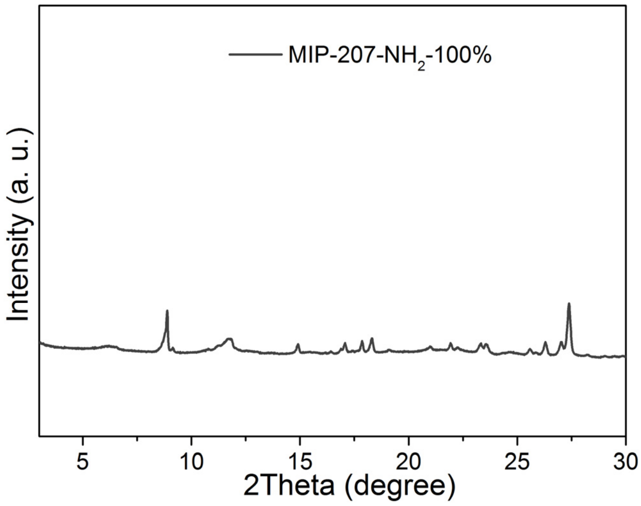

Figure S2. PXRD pattern of MIP-207-NH_2-100%.

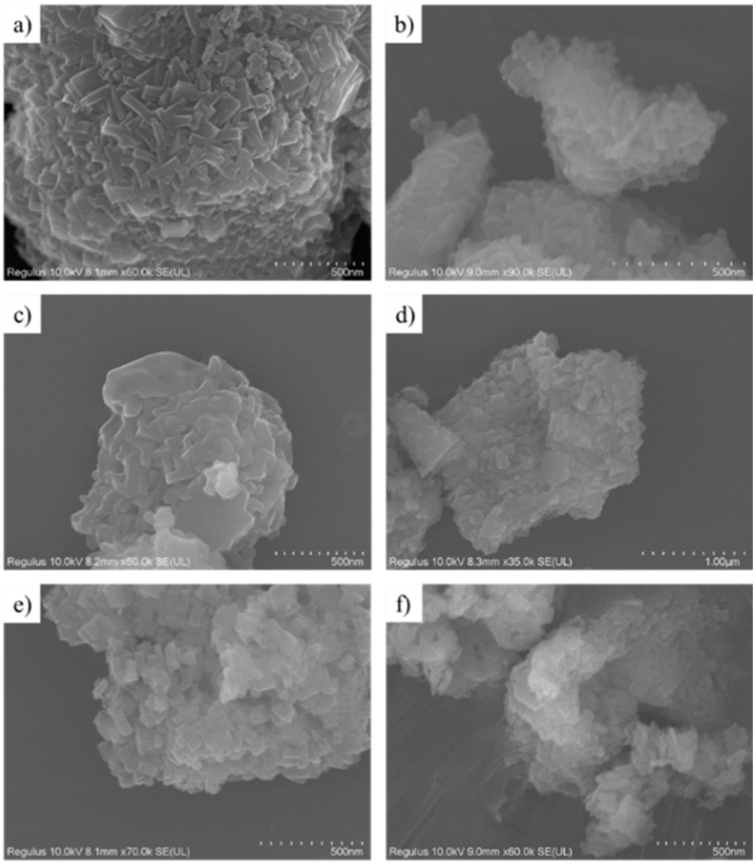

Figure S3. SEM images of (a-b) MIP-207, (c) MIP-207-NH_2-15%, (d) MIP-207-NH_2-25%, (e) MIP-207-NH_2-50%, (f) MIP-207-NH_2-60%.

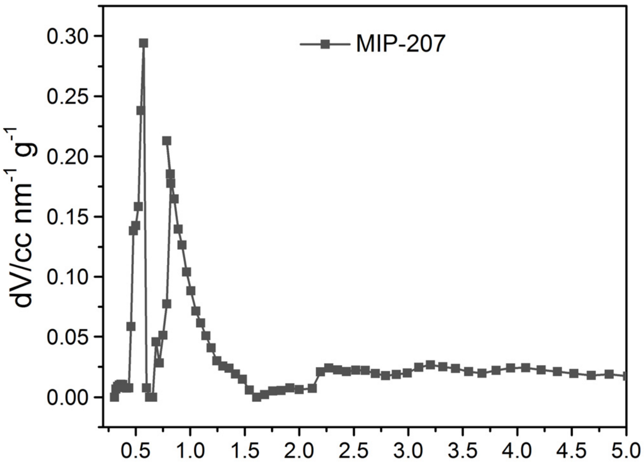

Figure S4 The pore size distribution curves of MIP-207.

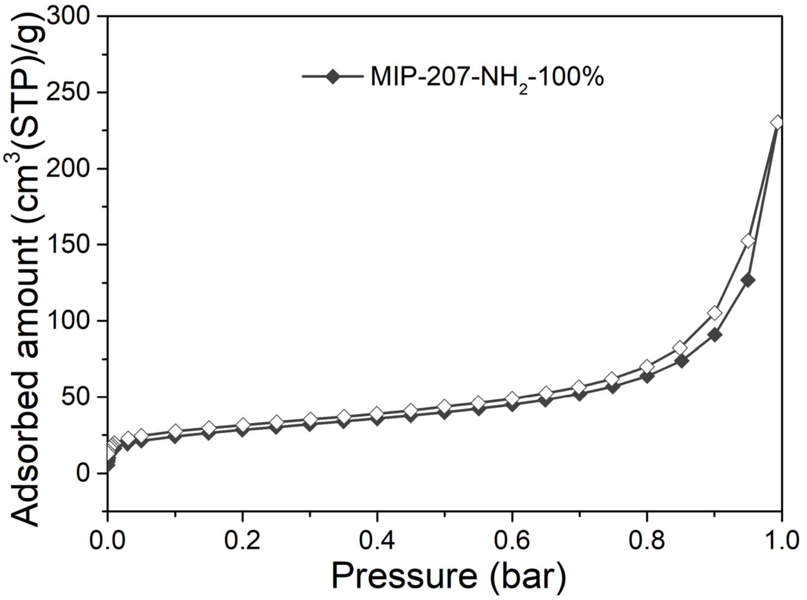

Figure S5 N_2 adsorption and desorption isotherms of MIP-207-NH_2-100%.

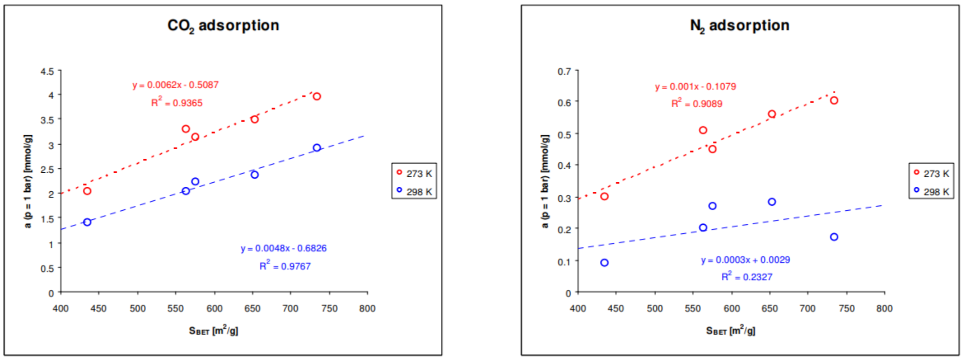

Figure S6 The relationship of between CO_2/N_2 adsorption and specific surface area of samples.

Table S1. The BET data of MIP-207-NH_2-100%.

Samples	BET area (m^2 g^{-1})	Micropore area (m^2 g^{-1})	Total pore volume (cm^3 g^{-1})	Micropore volume ($cm^3 g^{-1}$)
**MIP-207-NH_2-100%**	102	17	0.36	0.01

Reference

[1] S. Furmaniak, S. Koter, A.P. Terzyk, P.A. Gauden, P. Kowalczyk, G. Rychlicki, New insights into the ideal adsorbed solution theory, Phys. Chem. Chem. Phys., 17 (2015) 7232-7247.

[2] Z. Hu, M. Khurana, Y.H. Seah, M. Zhang, Z. Guo, D. Zhao, Ionized Zr-MOFs for highly efficient post-combustion CO 2 capture, Chem. Eng. Sci., 124 (2015) 61-69.

[3] R. Zhong, X. Yu, W. Meng, J. Liu, C. Zhi, R. Zou, Amine-Grafted MIL-101(Cr) via Double-Solvent Incorporation for Synergistic Enhancement of CO_2 Uptake and Selectivity, ACS Sustain. Chem. & Eng., 6 (2018) 16493-16502.
